# Practical application of precision oncology in adult onset craniopharyngiomas

**DOI:** 10.3389/fendo.2024.1488958

**Published:** 2024-11-20

**Authors:** Chandrima Biswas, Guilherme Mansur, Kyle C. Wu, Daniel M. Prevedello, Luma Ghalib

**Affiliations:** ^1^ Department of Neurologic Surgery, The Ohio State University Wexner Medical Centre, Columbus, OH, United States; ^2^ Division of Endocrinology, Diabetes and Metabolism, Department of Internal Medicine, The Ohio State University Wexner Medical Centre, Columbus, OH, United States

**Keywords:** targeted therapy, craniopharyngioma, papillary, adamantinomatous, BRAF, MEK inhibitors

## Abstract

**Introduction:**

Craniopharyngiomas (CPs) are benign and rare tumors found in adults. Their location close to vital neurovascular structures makes traditional treatment modalities (surgery and radiation) challenging and potentially fraught with morbidity. The 2021 WHO classification has divided what was previously considered two subtypes of craniopharyngioma into separate entities. Identification of specific molecular driver mutations in each type- BRAF V600E in papillary craniopharyngiomas (PCP) and CTNNB1 in adamantinomatous craniopharyngiomas (ACP) has resulted in a paradigm shift in the management of adult CPs.

**Methods:**

In this study, we describe our experience in treating PCPs with targeted therapy and highlight nuances in management accounting for current evidence. This review also explores the current scope and application of precision oncology in adult CPs including the experience with ongoing trials and prospects for future research.

**Results:**

The high prevalence of targetable mutation in cases of PCP and the efficacy of BRAF inhibitors alone or in combination with MEK inhibitors has improved the disease control in these patients. In the current scenario, while surgery is warranted to obtain histopathological diagnosis, radical resection and its associated risks can be avoided. In case of ACPs, dysregulation of multiple pathways has been implicated. This has prompted the use of a variety of targeted therapies with inconsistent outcomes. The results of ongoing and future trials may define its role in management.

**Conclusion:**

Precision oncology is a promising addition to the treatment armamentarium of adult CPs.

## 1 Introduction

Craniopharyngiomas (CPs) are rare tumors constituting 1.2–4.6% of all brain tumors with an incidence rate of 0.5–2.5 new cases per 1 million people (1). Although histologically benign, their close proximity to adjacent structures especially, the hypothalamus, pituitary gland and the optic apparatus result in sub-optimal visual, cognitive and endocrinologic outcomes. Papillary and adamantinomatous CPs are two independent entities with papillary craniopharyngiomas (PCP)more frequently found in adults than children and predominantly present as solid tumors without calcifications. Majority of these tumors (95%) are driven by BRAF V600E mutation (2). Adamantinomatous tumors (ACPs) are more prevalent than papillary tumors in both adult and paediatric populations. They more frequently harbor cystic changes and are often calcified (90% cases) (3). These tumors are often locally infiltrative and driven by the CTNNB1 mutations (2). With the identification of targetable mutations, craniopharyngiomas, in particularly PCPs have the potential for improved disease control with minimal surgical morbidity.

Testing for genetic mutations in craniopharyngioma has practical implications. The availability of kinase inhibitors and immunotherapeutic agents, and the reported radiologic and clinical response, will have dramatic implications on the management of craniopharyngiomas.

The article will review a step-by-step management of how a positive BRAF V600E mutation status altered the course of treatment and outcome of two clinical cases of papillary craniopharyngiomas.

## 2 Our experience using genetic testing and targeted therapy for papillary craniopharyngioma

### 2.1 Case 1

**Figure 1:**
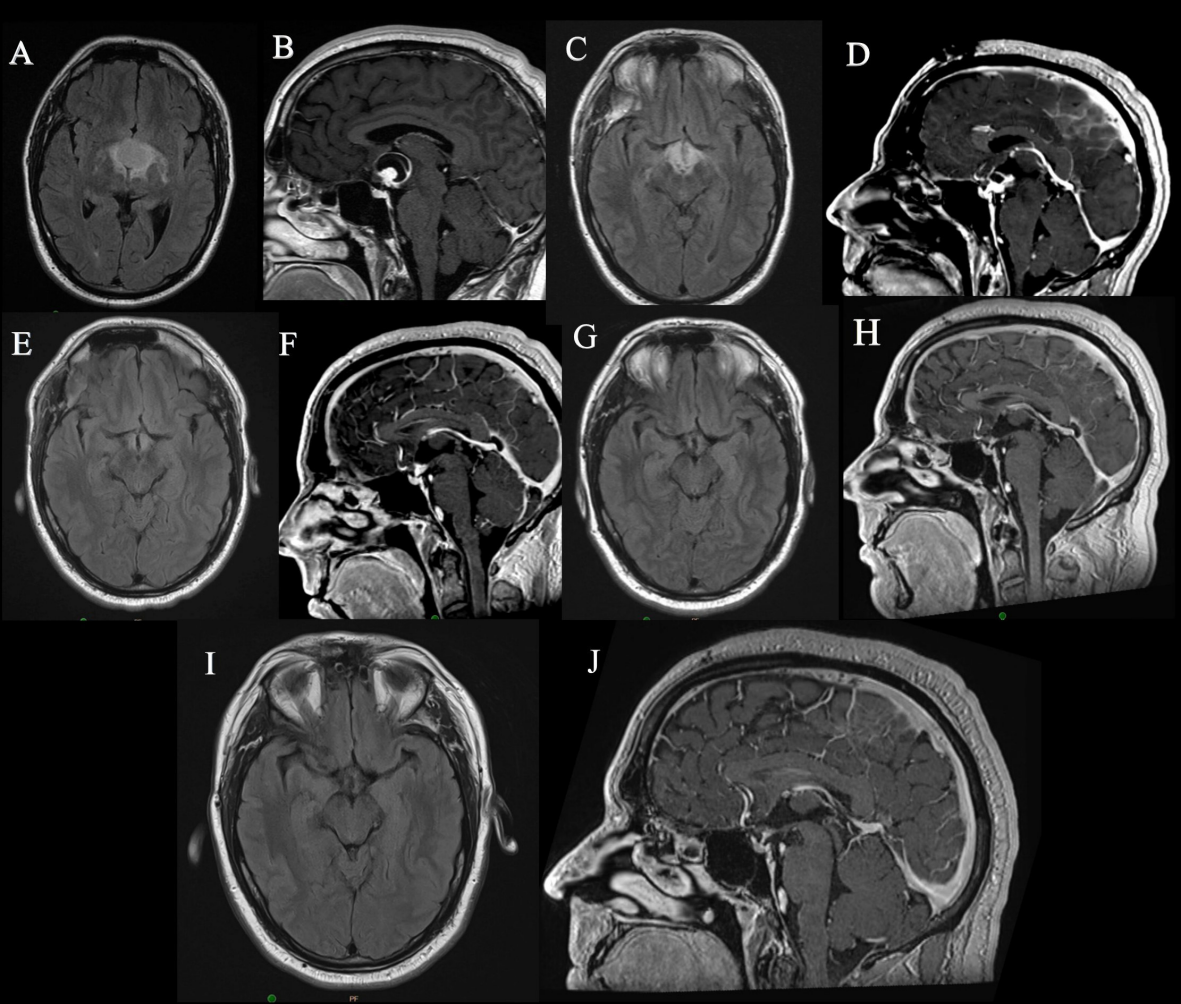
MRI images of Case 1: **(A, B)** Preoperative MRI images, **(C, D)** Immediate postoperative MRI images showing residual tumor, **(E, F)** MRI images after 1 month of dabrafenib, trametinib therapy showing significant reduction in size of tumor, **(G, H)** MRI images after 2 years of therapy, **(I, J)** MRI images obtained in last follow up, 5 years since surgery and 3 years since cessation of therapy.

A 44-year-old male presented with fatigue and weight gain. On examination, he was found to have features of hypogonadism and diabetes insipidus. His hormonal evaluation was significant for central hypothyroidism and hypogonadism. He was started on thyroxine, desmopressin and testosterone supplementation. His brain magnetic resonance imaging (MRI) revealed a suprasellar cystic lesion with an associated nodule centred in the region of the anterior third ventricle and hypothalamus and computed tomography (CT) scan did not show any calcification. Based on these findings, it was suspected to be a papillary craniopharyngioma (**Figures 1A, B**) and the patient underwent a right fronto temporal craniotomy. Intraoperatively, the cyst wall was found to be integrated with the tissue from the hypothalamus and the posterior aspect of the optic chiasm, hence the decision was made to not resect this portion of tumor. Postoperatively, the patient had no neurological deficit and was continued on corticosteroid, thyroid, testosterone and desmopressin supplementation. His histopathology was suggestive of papillary craniopharyngioma BRAFV600E mutation positive and residual disease was noted on the MRI as expected (**Figures 1C, D**). Considering the growing body of evidence demonstrating excellent response of BRAF/MEK inhibitors in tumors with this mutation, the decision to initiate therapy with dabrafenib/trametinib rather than to pursue radiotherapy (RT) was made by the tumor board. He was started on dabrafenib 150 mg twice daily and trametinib 2 mg once daily for 30 days each cycle. A routine MRI after the first cycle showed a dramatic reduction in the size of the tumor as shown in (**Figures 1E, F**). He underwent routine evaluation by a dermatologist, ophthalmologist and also underwent routine liver and renal function tests and echocardiography to monitor for adverse events.

After 7 cycles of treatment, he developed cardiotoxicity with reduced cardiac ejection fraction which was attributed to trametinib. He was continued on single agent dabrafenib 150 mg twice daily for 24 months at which time he elected to stop treatment During this time his tumor remained stable (**Figures 1G, H**). His MRI continues to demonstrate stable disease three years after stopping treatment (**Figures 1I, J**). During his treatment he developed multiple hyperkeratotic lesions on the forearms which monitored.

The key nuances of this course of treatment was- a) the decision to treat residual tumor with targeted therapy, rather than radiation to avoid injury to his endocrinologic function b) the selection of drug or drug combination, c) surveillance plan for both response to treatment and adverse events d) dose reduction during treatment when adverse events occur and e) cessation of treatment in the presence of continued disease stability. All these are questions, the multidisciplinary clinical teams need to navigate during the treatment plan as will be highlighted in the discussion.

### 2.2 Case 2

**Figure 2:**
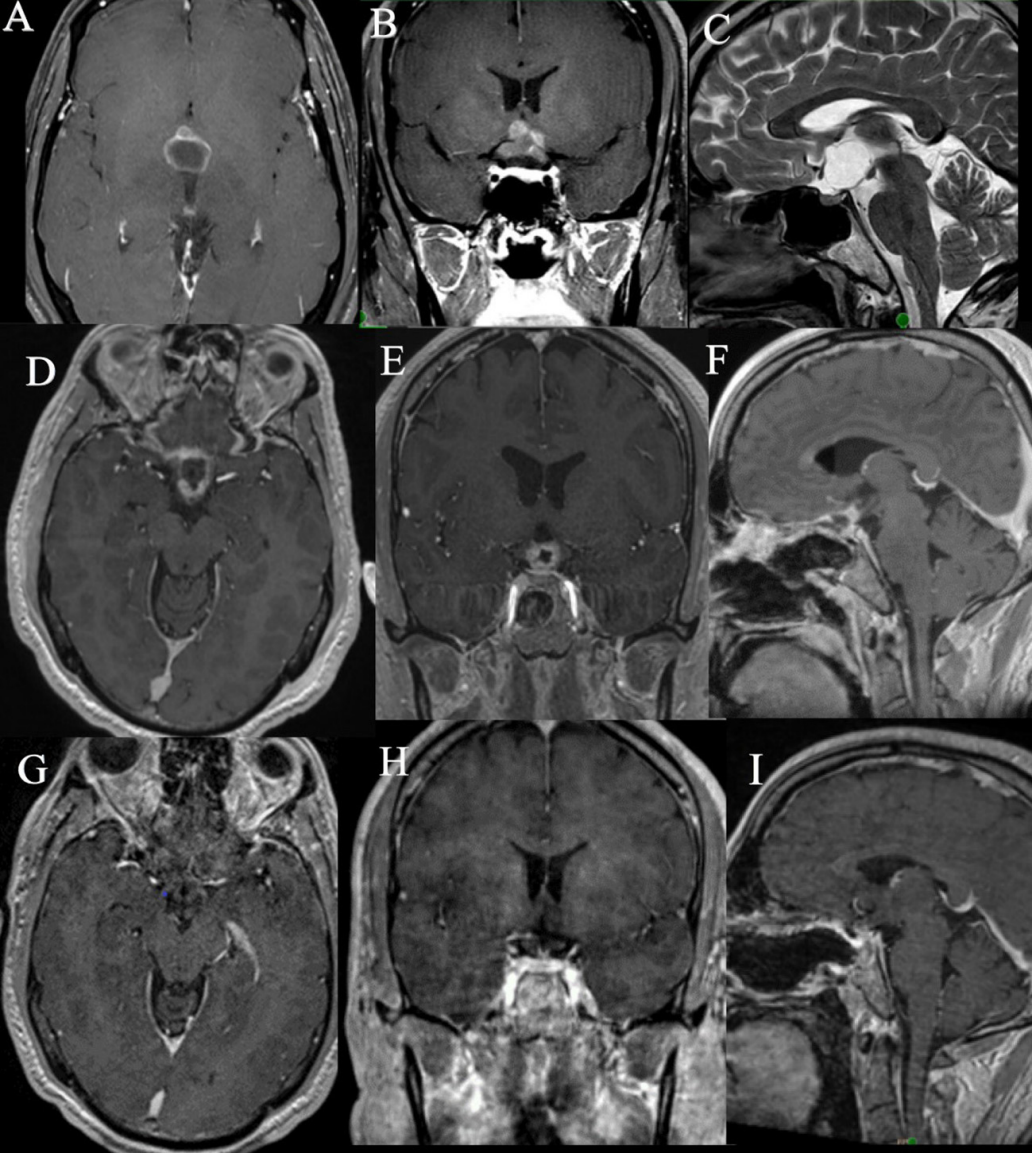
MRI images of Case 2: **(A–C)**. Preoperative MRI images, **(D–F)**. Immediate postoperative MRI images showing the residual tumor, **(G–I)**. MRI images obtained after 19 months of dabrafenib, trametinib therapy showing significant reduction in size of tumor.

A 40 year old gentleman presented with bilateral visual blurring, headaches and weight gain. Further evaluation showed bilateral visual field defects with bitemporal inferior quadrant defects. A brain MRI showed a large suprasellar cystic lesion with a small nodular enhancing component compressing the optic apparatus; on CT scan there were no calcifications (**Figures 2A–C**). Neuroradiology review and tumor board discretions suggested a likely PCP. His preoperative endocrinological evaluation was suggested diabetes insipidus which was managed with low dose desmopressin. He underwent resection of the lesion via an endoscopic endonasal trans-tubercular approach. The cyst was decompressed, and majority of the nodular component and the posterior cyst wall was resected, leaving behind a tiny portion of the solid part adherent to the surface of the pituitary stalk and the optic chiasm. Since it was likely that this tumor was a papillary craniopharyngioma and was adherent to critical structures, further resection was deemed unnecessary and unsafe. Postoperatively, visual symptoms and headaches improved. Postoperative MRI showed the presence of a residual nodular ring like enhancement along the resection margin as expected (**Figures 2D–F**).

The pathology confirmed papillary craniopharyngioma and on genomic sequencing revealed presence of the BRAF V600E mutation.

In view of the residual disease, the decision to give adjuvant therapy was determined by the multidisciplinary tumor board. Due to the proximity of the residual to the optic chiasm and hypothalamus, the possibility of adverse effects of radiation was considered, especially with the growing body of evidence favoring BRAF and MEK inhibitor therapy for such tumors. A detailed discussion of risks and benefits was held with the patient, and he chose to undergo BRAF/MEK- dabrafenib-trametinib therapy foregoing radiation. The patient was started on dabrafenib 150 mg twice daily and trametinib 2 mg daily for 30 days each cycle. He was regularly monitored with an echocardiography and by dermatology. His MRI after 3 weeks of therapy showed a significant reduction in the size of the nodular component. He has been on treatment for 19 months now and has reported no toxicity. He showed no signs of toxicity after 19 months of treatment. There was near total regression of residual disease as seen on the MRI (**Figures 2G–I**) and the patient did not require RT.

## 3 Discussion

### 3.1 Targeted therapy in BRAF V600E tumors

The BRAF alterations including BRAF V600E mutation is found in many cancers most notably malignant melanomas, anaplastic thyroid cancers and pediatric low grade gliomas. It leads to activation of the MAPK pathway which results in uncontrolled phosphorylation of downstream MEK and ERK, eventually leading to unregulated cell growth and differentiation. The United States Food and Drug Administration has approved use of the combination of BRAF and MEK inhibitors for BRAF V600E mutant malignant melanomas, anaplastic thyroid cancers, non-small cell lung cancers, pediatric low grade gliomas requiring systemic therapy and metastatic unresectable solid tumors as a tissue agnostic treatment. Recent molecular studies have identified BRAF V 600E mutations in 95% of patients with PCP (2). The prevalence of this mutation along with the dramatic response with BRAF/MEK inhibitor combination therapy in a recent phase 2 clinical trial- has dramatically changed the landscape of treatment of these tumors (4).

#### 3.1.1 Single agent Vs combination therapy

The rationale for using a combination treatment is to inhibit both upstream BRAF and downstream MEK, thus providing a synergistic effect. The BRAF inhibitor monotherapy has been associated with paradoxical activation of the MAPK pathway which has been linked to development of acquired resistance within 6-7 months of treatment (5, 6). It has also been associated with various side effects including secondary skin cancers (7, 8). Several randomised clinical trials have shown that using a combination of BRAF inhibitor and mitogen activated extracellular signal regulated kinase (MEK) inhibitor not only prevent or delay MAPK driven acquired resistance but also improves progression free survival and overall survival (9). However, these interpretations have been derived from trials of patients with malignant melanoma and although there are several case reports and series highlighting remarkable response to monotherapy and combination therapy in patients with PCP, there is no evidence to suggest that one is more effective or safer than the other.

#### 3.1.2 Adverse events

There are three BRAF inhibitors in use- vemurafenib, dabrafenib and encorafenib. Common side effects of BRAF inhibitors include rash, joint pains and fatigue. Excessive sensitivity to sunlight may occur with vemurafenib. Hyperkeratosis and dysesthesia can be seen during encorafenib monotherapy. There are three BRAF/MEK inhibitor combinations used for treatment of BRAF V600 E positive melanomas-1) vemurafenib/cobimetinib 2) dabrafenib/trametinib and 3) encorafenib/binimetinib. The common side effects of MEK inhibitors include rash, diarrhea, peripheral edema and fatigue. Acneiform dermatitis and papulopustular rash can occur with trametinib use. Nausea and vomiting have been reported following binimetinib (10).

The combination of these drugs does not however potentiate toxicity. In a recent metanalysis (10) looking into the adverse events with BRAF or MEK inhibition alone and in combination, it was seen that the combination of dabrafenib/trametinib is associated with a significantly lower rates of grade 3- 5 adverse events (43%) than dabrafenib alone (50%). Amongst the combination therapy, incidence of grade 3-5 toxicity was higher with vemurafenib-cobimetinib (72%) and encorafenib-binimetinib (68%) than dabrafenib-Trametinib (43%). Grade 1- 2 side effects are common in all the three combinations and included- arthralgia, alopecia, asthenia, fatigue, rash, headache and pyrexia. Onset of headache, pyrexia and anorexia was more frequently found in the combination of dabrafenib/trametinib. The incidence of arthralgia, hypertension and decreased EF, skin rashes, diarrhea and elevated liver enzymes was highest when vemurafenib/cobimetinib was used in combination.

While different drugs and drug combinations have a different side effect profile, the choice of targeted agent, however, is tailored on case-by-case basis depending on the pre-existing conditions of the patient and availability. In our cases, a decision to start dabrafenib/trametinib therapy was made after careful consideration and discussion with the patients, multiple providers, and their caregivers along with the details of drug availability, therapy cost and insurance coverage.

### 3.2 Routine surveillance plan

A detailed treatment and surveillance plan needs to be created not only to record response to treatment using serial imaging but also to identify early onset of adverse events. Our patient underwent MRI scans every 4 weeks till stability of the lesion was documented and thereafter every 2 months. He underwent renal and liver function tests and complete blood count before every cycle and also a 2D echocardiography every 2-3 months. Once cardiotoxicity was detected with reduced ejection fraction, therapy was stopped until repeat 2D echocardiography in a month demonstrated reversal of EF back to baseline. Therapy was continued with dabrafenib monotherapy without dose reduction. The patient also underwent routine ophthalmological evaluation and screening for dermatologic and pulmonary problems. During his course of treatment, he developed multiple hyperkeratotic lesions over his arms, and seborrheic keratosis which was manged conservatively.

### 3.3 Duration of therapy

In the recent phase 2 study evaluating the efficacy of BRAF/MEK inhibitor combination vemurafenib-cobimetinib in patients with PCPs, 12 of the 16 patients (75%) had grade 3 adverse events during treatment with 5 patients (31%) discontinuing treatment due to toxic effects. This high incidence of adverse events although similar to other studies is problematic for long term daily therapy for an indolent and benign tumor. Therefore, there needs to be a balance between risk of adverse events and tumor progression when deciding the duration of treatment.

Our patients showed near complete response to therapy and were continued on dabrafenib therapy for a total of 23.5 months (case 1) and combination of dabrafenib/trametinib for 19 months (case 2). Both patients had stable disease in their last follow up imaging (12 months and 24 months after stopping therapy respectively). The question of whether a shorter duration of treatment could suffice needs to be answered in larger clinical trials. Evidence from several case reports suggests that BRAF/MEK inhibitors produce efficient tumor shrinkage in the first 3 months of therapy (11). In the phase 2 trial using vemurafenib- cobimetinib for PCP 18.7% patients had recurrence after a median of 8 treatment cycles. Evidence of primarily cystic recurrence after therapy in previously mixed solid cystic tumors was noted in few cases (12, 13) even when the solid tumor showed stability. This underscores the importance of evaluating the duration of treatment for individual drug and drug combinations and also take into consideration the tumor consistency that may affect recurrence.

### 3.4 Need for surgery or radiation post therapy

Currently all reported use of BRAF inhibitors in BRAF V600 E positive PCP has shown a positive response in reducing tumor size. Most patients show subjective improvement in vision (14–16) with many having a stable residual disease at the end of treatment (12–22). The course of treatment following achievement of stable disease is a question that needs to be answered. While we managed our patients with serial clinico-radiological surveillance after treatment, targeted therapy has been proposed as a form of neo adjuvant therapy before definitive treatment (radiotherapy or surgery) (13, 16, 17, 20) or concurrent to radiation (23). With the significant size reduction achieved with BRAF/MEK inhibitors in most of these cases, whether definitive surgery or radiation can be avoided, needs to be addressed. However, they (surgery and/or radiation) may be useful in patients unable to tolerate the drugs or in cases of recurrent or progressive disease (4).

### 3.5 Role of surgery in the current era

The premise of this targeted therapy relies on the detection of BRAF V600E mutation. While surgery offers the gold standard tissue diagnosis, other non-invasive approaches for molecular diagnosis that may be promising include BRAF mutation analysis in cell free (cf) DNA in plasma, serum and CSF, as well as MRI-based radiomics. There is extensive literature highlighting the utility of blood based liquid biopsies in several extracranial solid tumors such as melanoma, breast, lung and colorectal cancers (24–27), however its utility in primary brain cancers has been impeded by low detection rates compared with systemic solid tumors presumably due to less cfDNA shedding into systemic circulation (28). In a recent study (29) digital droplet PCR (ddPCR) detected BRAF V600 E alterations in 60% baseline samples of cf DNA in plasma of patients with high grade gliomas. Serial plasma evaluation may help track this mutation in cf DNA and correlate with disease progression before radiological confirmation. Development of radiomics based models incorporating features such as texture, intensity, shape and wavelength in addition to location of tumor have shown promise in diagnosing BRAF V600E mutation (30). However larger studies may be needed before routine clinical applications in adult craniopharyngiomas.

At this time, surgical intervention remains standard of care for diagnostic purposes. The goal of surgical intervention is primarily to obtain pathologic diagnosis that will guide surgical treatment. This can often be achieved by obtaining a frozen pathologic specimen to differentiate between PCP and ACP. In cases where intraoperative pathology reveals PCP, a more conservative approach to surgery should be favored given the effectiveness of targeted treatments. In cases of adamantinomatous tumor maximum safe resection plays a primary role in the management of these tumors allowing diagnosis and possibility of complete cure.

### 3.6 Current scenario of precision medicine in adamantinomatous craniopharyngioma

In patients with ACP, recurrence rates range from 23-50% even after complete resection (31–33). Whereas, subtotal resection followed by RT yields comparable rates of recurrence and survival (32, 34). This underscores the need for effective medical therapy to halt the progression of disease and avoid RT. Studies on ACPs have revealed dysregulation of multiple potentially targetable molecular pathways. Dysregulation of WNT/B-catenin pathway, which is necessary for the organ formation, maintaining the stem cells and controlling gene transcription is found in 57-96% of ACPs (2, 35), however, targeting this pathway may be associated with unwanted off-target effects (36) due to its role in homeostasis and differentiation.

In a phase II trial, interferon 2α (IFN-2α) (known to induce apoptosis in skin cancer) was used in patients with progressive or recurrent ACPs. However, it demonstrated progressive disease (PD) in several cases (37). Yeung et al. (38) explored the efficacy of pegylated IFN-α-2b, reporting a complete remission (CR) in one patient and partial remission (PR) in others. Elevated concentrations of proinflammatory mediators, including interleukin 6 (IL-6), IL-8, IL-10 in CF tumor tissue prompted the use of tocilizumab and bevacizumab, achieving a decrease in cystic disease after initiation of combination therapy (39). Nussbaum et al. (40) utilized dabrafenib and trametinib, showing a CR with over 95% tumor reduction after 21 months of treatment.

Due to evidence of dysregulation of the MAPK/ERK pathway in ACP (41), the role of MEK inhibitors has been explored. In our own experience treating a residual ACP with binimetinib, the patient developed PD 11 months after therapy and was severely symptomatic from the disease and the adverse effects. Over-expression of vascular endothelial growth factor (VEGF) has been demonstrated in ACPs (42) and positive response to anti VEGF antibody (bevacizumab) as monotherapy and combination with tocilizumab has been reported in recurrent and progressive ACPs (39, 43). Thus, the variety in targeted therapies highlights the ongoing efforts to identify effective targeted interventions for adamantinomatous craniopharyngiomas. This variability in treatment responses underscores the need for a better understanding of targetable mutations to inform precision treatment (44).

## 4 Conclusion

Targeted therapy is a promising adjunct in the treatment of adult CPs. In PCPs it has shown to reduce tumor burden without the morbidity associated with surgery and RT. Further research is needed to establish an optimal combination of therapeutic modalities to tailor the treatment as per the individual needs.
